# Optimizing Predictive Performance of Bayesian Forecasting for Vancomycin Concentration in Intensive Care Patients

**DOI:** 10.1007/s11095-020-02908-7

**Published:** 2020-08-23

**Authors:** Tingjie Guo, Reinier M. van Hest, Laura B. Zwep, Luca F. Roggeveen, Lucas M. Fleuren, Rob J. Bosman, Peter H. J. van der Voort, Armand R. J. Girbes, Ron A. A. Mathot, Paul W. G. Elbers, Johan G. C. van Hasselt

**Affiliations:** 1 Department of Intensive Care Medicine | Research VUmc Intensive Care (REVIVE) | Amsterdam Cardiovascular Sciences (ACS) | Amsterdam Medical Data Science (AMDS), Amsterdam UMC, Vrije Universiteit Amsterdam, Amsterdam, The Netherlands; 2 Department of Pharmacy, Amsterdam UMC, University of Amsterdam, Amsterdam, The Netherlands; 3 Division of Systems Biomedicine and Pharmacology, Leiden Academic Centre for Drug Research (LACDR), Leiden University, Leiden, The Netherlands; 4 Mathematical Institute, Leiden University, Leiden, The Netherlands; 5 Intensive Care Unit, OLVG Oost, Amsterdam, The Netherlands

**Keywords:** bayesian forecasting, ICU, MAP, NONMEM, TDM, vancomycin

## Abstract

**Purpose:**

Bayesian forecasting is crucial for model-based dose optimization based on therapeutic drug monitoring (TDM) data of vancomycin in intensive care (ICU) patients. We aimed to evaluate the performance of Bayesian forecasting using maximum a posteriori (MAP) estimation for model-based TDM.

**Methods:**

We used a vancomycin TDM data set (*n* = 408 patients). We compared standard MAP-based Bayesian forecasting with two alternative approaches: (i) adaptive MAP which handles data over multiple iterations, and (ii) weighted MAP which weights the likelihood contribution of data. We evaluated the percentage error (PE) for seven scenarios including historical TDM data from the preceding day up to seven days.

**Results:**

The mean of median PEs of all scenarios for the standard MAP, adaptive MAP and weighted MAP method were − 7.7%, −4.5% and − 6.7%. The adaptive MAP also showed the narrowest inter-quartile range of PE. In addition, regardless of MAP method, including historical TDM data further in the past will increase prediction errors.

**Conclusions:**

The proposed adaptive MAP method outperforms standard MAP in predictive performance and may be considered for improvement of model-based dose optimization. The inclusion of historical data beyond either one day (standard MAP and weighted MAP) or two days (adaptive MAP) reduces predictive performance.

**Electronic supplementary material:**

The online version of this article (10.1007/s11095-020-02908-7) contains supplementary material, which is available to authorized users.

## Introduction

Therapeutic drug monitoring (TDM) concerns the measurement of drug concentrations in patients to optimize dosing schedules in individual patients and is commonly used in treatment optimization of patients of intensive care unit (ICU). Population pharmacokinetic (PK) models are regularly used to derive optimized dosing regimens based on TDM data. These model-based dosing regimens are guided by Bayesian forecasting through maximum a posteriori (MAP) estimation. Using MAP estimation, individual PK parameters are estimated using a previously developed population PK model based on collected historical drug concentration TDM data of a patient who has received the drug. These individual PK parameters in turn can be used to perform Bayesian forecasting to predict the prospective concentrations to further derive a dosing schedule that meets therapeutic concentration targets associated with efficacy or toxicity.

There is currently a lack of consensus or guidelines regarding the use of historically collected TDM PK data for Bayesian forecasting. Given that TDM is typically associated with sparse PK samples, inclusion of all available TDM data may arguably lead to optimal use of data and as a consequence individual PK parameter estimates closer to the true value in a patient. However, in ICU patients, rapid alterations in organ functions associated with PK might also actually lead to increased bias in the current PK parameter estimates if too much historical TDM data is included (1). In ICU patients it is therefore unclear if and how such historical TDM data should be included for Bayesian forecasting, and, if current MAP-based approaches optimally make use of available historical data in estimating individual PK parameters. In the current analysis we aimed to address these questions using a representative TDM data set for vancomycin in ICU patients. Vancomycin forms a cornerstone antibiotic agent for treatment of sepsis associated with gram-positive infections in the ICU. In the ICU population, vancomycin usually shows large inter-individual variability as well as large intra-individual variability in PK (2,3). TDM is routinely performed for vancomycin in the ICU due to its narrow therapeutic window and rapid alterations in organ function that could lead to changes in PK (3–6). As such, vancomycin represents a relevant paradigm drug to study the optimization of model-based TDM approaches.

In this analysis we propose and evaluate two methods of MAP estimation for model-based dose optimization using TDM data. We will refer to the newly proposed methods as adaptive MAP and weighted MAP. The proposed methods are compared to standard MAP estimation.

## Materials and Methods

### Data

A retrospective vancomycin TDM data set collected in ICU patients was used for this analysis, containing vancomycin concentration-time data, vancomycin dosing histories, and patient demographics, collected from two hospitals in The Netherlands. The patients from one hospital received vancomycin with a loading dose of between 1000 mg and 2000 mg followed by doses of 1000 mg twice a day until doses were adjusted at the discretion of the treating clinician. The patients from the other hospital received vancomycin with a loading dose, followed by continuous infusion. Both loading doses and continuous infusion doses were individualized based on the advice from the in-house-developed model-based TDM software AutoKinetics (7). The infusion duration time ranged from one to two hours for non-continuous infusion and was 24 h for continuous infusion. Blood samples were obtained on average twice or three times every week mainly at trough level, i.e. prior to the next dose. A serum creatinine measurement was also measured for each sample. The creatinine clearance of the patients were calculated by default based on the MDRD formula, provided that the commonly seen formulae for calculating glomerular filtration rate were expected to perform similarly in ICU patients (8,9). We excluded the patients if plasma concentration samples were only available for a single day of treatment since model predictions cannot be validated for such patients. As a result, a data set consisting of 2435 of concentration data points from 408 patients was used (Table I).

**Table I:** Characteristics of the Patients in this Study

Characteristics	Mean ± SD
No. of patients	408 (CI = 372)
No. of data points	2435
Samples/Patient	6
Sampling frequency	2 to 3 samples/week
Loading dose	1000 mg*
Following dose	1000 mg twice a day*
Infusion duration	Ranging from 1 to 2 h**
Age (years)	67 ± 12
Male (%)	63%
Weight (kg)	84 ± 18
CrCL (ml/min/1.73m^2^)	66.5 ± 53.1

CI, continuous infusion

CrCL, creatinine clearance calculated according to MDRD formula (8)

*Both loading dose and following dose may be adjusted according to the advice from AutoKinetics (7)

**Infusion duration time was adjusted at the discretion of treating nurses and was always 24 h for continuous infusion

### Population Pharmacokinetic Model

A previously published one-compartmental population PK model of vancomycin by Roberts *et al*. 2011 (Table II) was used to perform both the MAP estimation and Bayesian forecasting (10). The model has been validated in our own ICU population, with the same data as used in this study (11).

**Table II:** The Vancomycin PopPK Model (10)

Component	Equation
Pharmacokinetic parameters	CL (L/h) = 4.58·CrCL/100·*e*^*η*^_1_ V (L) = 1.53 ·WGT·*e*^*η*^_2_
Inter-individual variability	*η*_1_ ~ *N*(0, ${\omega}_1^2$) and *η*_2_ ~ *N*(0, ${\omega}_2^2$) *ω*_1_= 0.389 and *ω*_2_ = 0.374
Residual errors	Obs = Pred·(1 + *ε*_1_) + *ε*_2_ *ε*_1_~ *N*(0, ${\sigma}_1^2$) and *ε*_2_ ~ *N*(0, ${\sigma}_2^2$) *σ*_1_ = 0.199 and *σ*_2_ = 2.4 (mg/L)

CL, clearance; V, volume of distribution; CrCL, creatinine clearance in ml/min; WGT, body weight in kg; Obs, observed concentration; Pred, predicted concentration

### MAP Estimation

**Fig. 1:**
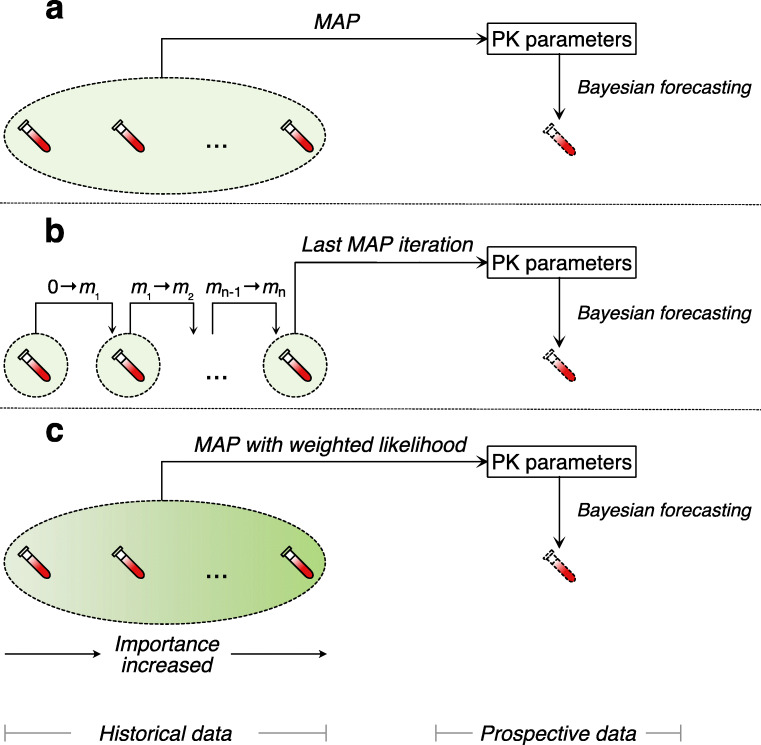
Schematic diagram of methods of MAP estimation used in the study. Standard MAP executes estimation once using all historical TDM data (**a**); Adaptive MAP executes estimation iteratively using each segment of historical TDM data with updated prior mean by posterior mode from its preceding iteration and repeats until the last iteration, i.e. 0 to *m*_1_, …, *m*_n-1_ to *m*_n_ (**b**). Weighted MAP executes estimation once using all historical TDM data with weighted importance (likelihood) of each segment of data during the estimation (**c**).

Data splitting was needed in order to evaluate different MAP methods. The data set was split into segments where each segment contained the concentration data of a single day. Thus, one segment corresponded to one day which contained one trough sample. For each patient, we estimated individual PK parameters based on one to up to seven consecutive segments of data to predict the concentration data of the succeeding segment. Hence, a segment of data was regarded as either historical data if used for MAP estimation, or as prospective data if used to validate the model predictions. We compared three estimation methods: standard MAP, adaptive MAP and weighted MAP. Figure 1 depicts the conceptual differences between these methods, and are defined further in below.

#### Standard MAP

Given a model with random-effect (of clearance (CL) and volume of distribution (V)) parameter vectors **η**, the standard MAP estimation constructs a posterior density distribution as follows (12):

1 $$ z\left(\boldsymbol{\upeta} \right)=f\left(\boldsymbol{\upeta} |\mathbf{Y}\right)g\left(\boldsymbol{\upeta} \right) $$

where *f*(**η**|**Y**) is the likelihood of **η** given concentration observation vector of **Y.** *g*(**η**) denotes the prior density distribution of **η**, which followed a multi-normal distribution informed by the vancomycin population PK model (Table II). *z*(**η**) is the posterior density distribution that MAP estimation aims to maximize so that the mode (maximum) value of *z*(**η**) is the final estimate of **η**.

#### Adaptive MAP

The adaptive MAP shared a similar idea of model predictive control theory (13). The estimation was executed iteratively, in which each iteration used one segment of the historical data. For the 1st iteration, we executed MAP estimation using the earliest one segment of data to obtain the random-effect parameters (posterior mode) which were denoted as m_1_. For the 2nd iteration, the means of the variance of the random-effect parameters (prior mean), which are 0 under usual assumptions, were substituted by m_1_. Then the MAP estimation was executed using the next segment of data. By this way, the prior means of each iteration (except for the first one) were updated by the posterior modes of the last iteration. Such process was repeated until only one segment of data was left, which was used to validate model predictions (Fig. 1**b**).

#### Weighted MAP

Like the standard MAP estimation, the weighted MAP estimation was executed once using all historical data (Fig. 1**c**). However, the contribution of each segment of historical data to the total likelihood was weighted according to the following functions:

2 $$ z\left(\boldsymbol{\upeta} \right)=f{\left(\boldsymbol{\upeta} |\mathbf{Y}\right)}^{w\left({\mathbf{Y}}_s\right)}g\left(\boldsymbol{\upeta} \right) $$

3 $$ w\left({\mathbf{Y}}_s\right)={\left(\frac{\Delta {T}_{Ref}}{{\Delta T}_{{\mathbf{Y}}_s}}\right)}^{\alpha } $$

where *w*(**Y**_*s*_) is a weighting function for the likelihood of the parameter to be estimated given observation **Y**_*s*_, which is a subset of **Y** e.g. the concentration data at a particular day prior to the data to be predicted. ${\Delta T}_{{\mathbf{Y}}_s}$ stands for the time distance in days between the historical data **Y**_*s*_ and the data to be predicted. Δ*T*_*Ref*_ and *α* are both weighting factors. Δ*T*_*Ref*_ is the reference day, defined as the cutoff value of the time distance where *w*(**Y**_*s*_) is 1, i.e. where Δ*T*_*Ref*_ equals ${\Delta T}_{{\mathbf{Y}}_s}$. *α* is the unitless effect size of the weighting function *w*(**Y**_*s*_) on the likelihood *f*(**η**|**Y**). For the sake of simplicity, we only tested a number of integer combinations of Δ*T*_*Ref*_ and *α* including integers from 1 to 7 for Δ*T*_*Ref*_, and integers 1 to 5 for *α*. By this method, the individual likelihood of each data point to the total likelihood was exponentiated by *w*(**Y**_*s*_) so that its importance for the MAP estimation was weighted.

### Evaluation of MAP Methods

**Fig. 2:**
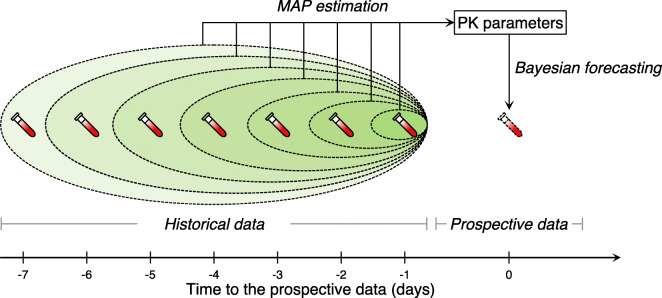
Schematic diagram of including historical data for MAP estimation and Bayesian forecasting of one patient.

The evaluation of the MAP methods was based on the use of different amounts of historical TDM data for each patient to estimate the PK parameter and predict a prospective drug concentration. To this end, a segment of data may be used as historical data for MAP estimation or as prospective data to validate model predictions in different scenarios. The prediction of prospective data was done using historical data from preceding one up to seven days (Fig. 2).

To evaluate predictive performance of each method, we calculated the difference between the observed and predicted vancomycin concentration, by calculating the percentage error (PE) as follows:

4 $$ {PE}_i=\frac{1}{n_i}\sum \limits_{j=1}^{n_i}\frac{{\hat{Y}}_{ij}-{Y}_{ij}}{Y_{ij}}\times 100\% $$

Here, *PE*_*i*_ denotes percentage error for *i*th subject; *n*_*i*_ equals to the number of forecasted concentration of *i*th subject; ${\hat{Y}}_{ij}$ and *Y*_*ij*_ represent the predicted and observed value of the *j*th prospective concentration of *i*th subject, respectively. We normalized the PE to the number of data points of the patient, to equalize the weight of each patient in the results. The accuracy and the precision were measured as the median and the interquartile range (IQR) of the PE, respectively. The results of PE were also visualized using a bar plot for standard MAP and adaptive MAP methods, and a heat map for weighted MAP method.

### Implementation

We executed the MAP estimation with all three methods using historical data to obtain individual PK parameters. We also estimated PK parameters using each segment of data set to explore the trend of the parameters’ change over time. Nonlinear mixed-effects modeling software (NONMEM, version 7.4.4; ICON Development Solutions, MD, USA) was used for both MAP estimation and Bayesian forecasting. The NONMEM code of the implementation of all methods is available as supplementary material. Data organization and visualization were carried out with R (version 3.6.0; R-project.org).

## Results

### Impact of the Methods of MAP Estimation

**Fig. 3:**
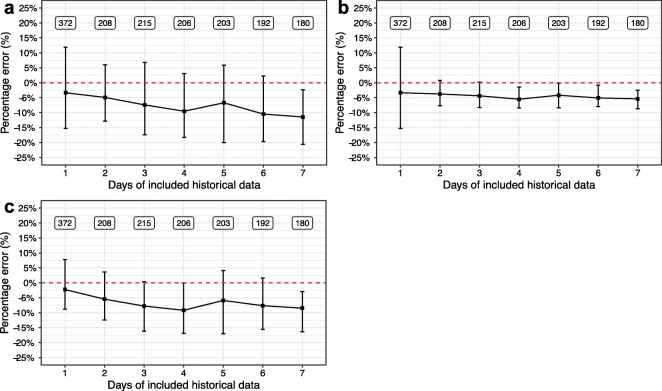
The percentage error of Bayesian forecasting using the standard MAP method (**a**), the adaptative MAP method (**b**), and the weighted MAP method using optimal weighting factors Δ*T*_*Ref*_=4 and *α*=2 (**c**). The squares are the median values, and the bars represent 25% and 75% quantile lines respectively. The labeled text is the number of patients that were included for the calculation.

**Fig. 4:**
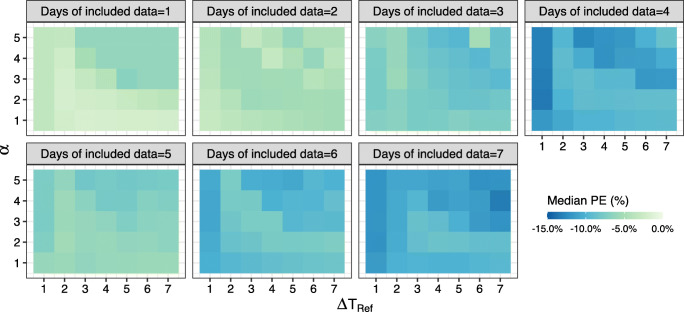
The percentage error of Bayesian forecasting using the weighted MAP method for all combinations of weighting factors Δ*T*_*Ref*_ and *α*. Δ*T*_*Ref*_ and *α* are both weighting factors. Δ*T*_*Ref*_ is the reference day, defined as the cutoff value of the time distance where *w*(**Y**_*s*_) is 1, i.e. where Δ*T*_*Ref*_ equals ${\Delta T}_{{\mathbf{Y}}_s}$. *α* is the unitless effect size of the weighting function *w*(**Y**_*s*_) on the likelihood *f*(**η**| **Y**).

The median PEs of including preceding one up to seven days of historical data for the standard MAP method ranged from −3.4% to −11.5% and for the adaptive MAP method, −3.4% to −5.4% (Fig. 3a, b). For the weighted MAP method, the median PEs ranged from −2.3% to −8.5% (using optimal values for weighting factors Δ*T*_*Ref*_=4, *α*=2 based on the mean and median values of median PEs that were closest to 0) (Fig. 3c). The adaptive MAP method thus outperformed the standard MAP method, whilst the effect of weighted MAP method was limited. We did not find a clear effect of the weighting factors Δ*T*_*Ref*_ and *α* (Fig. 4). The IQR of the PE for the standard MAP method and the weighted MAP method were both visibly wider than that for the adaptive MAP method, indicating the adaptive MAP method was most precise. An alternative error bar plot based on mean values and 95% confidence intervals is available in the supplementary material.

### Impact of the Number of Days of Included Historical Data

Including only one preceding day of historical data led to the most accurate Bayesian forecasting (Figs. 3 and 4). The PE worsened noticeably when including more historical data and using the standard MAP method and the weighted MAP method, while was consistent when using the adaptive MAP method (Figs. 3 and 4). The adaptive MAP method was therefore most robust to the included amount of historical data. The IQR of PE did not show clear relationship with the number of days of included historical data, regardless of the MAP method. However, the adaptive method resulted in the narrowest IQR which decreased markedly when using more than one preceding day of historical data (Fig. 3b). Thus, including the preceding two days of historical data for the adaptive MAP method guaranteed both accuracy and precision. Given the current setting of data splitting, including previous e.g. three segments of historical data can be also seen as including one segment of historical data which contains three days of concentration data. Hence, the similar results would be expected.

### Time Course of the Random-Effects Parameters

**Fig. 5:**
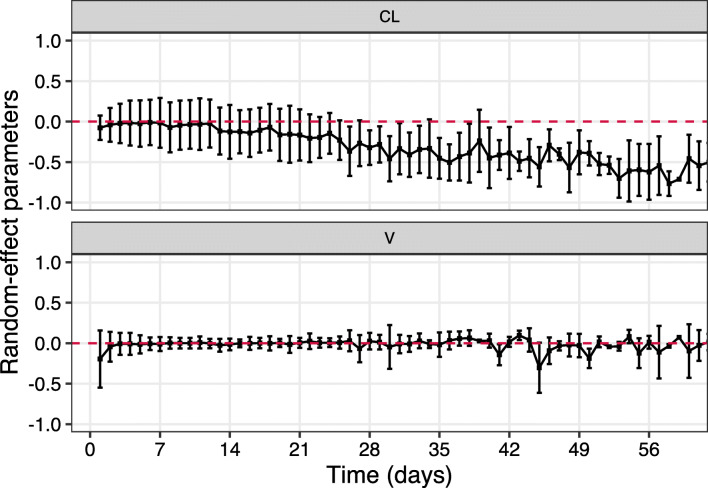
Time course of random-effects on PK parameters over time. CL, clearance; V, volume of distribution. The squares are the mean values, and the bars represent mean plus standard deviation and mean minus standard deviation lines respectively.

The random-effects of both PK parameters, CL and V, were estimated for each day (Fig. 5). The random-effect of CL showed a decreasing trend over time while that of V was rather stable. Such a result is in agreement with the observation that the PE values are all negative, since using a retrospective CL of a higher value would result in underprediction of the prospective data.

## Discussion

Adequate dosing is crucial for successful treatment of critically ill patients. Bayesian forecasting is probably the most commonly used approach in model-based TDM for ICU patients. There is unfortunately no evidence how Bayesian forecasting should be made properly. Our study investigated to optimize the use of historical TDM data in this special population.

Although potentially counter-intuitive, we found that including more historical TDM data can result in reduced forecasting accuracy. From the clinical perspective, this is not surprising since PK properties of ICU patients can change rapidly with disease progression. This means that older historical data contains outdated information that leads to bias in Bayesian forecasting. Thus, TDM samples that are drawn more closely to the ones to be forecasted in terms of time, are more likely to have better predictive abilities. Similar findings were reported in a previous study that if only a single sample was utilized, Bayesian predicted concentrations were less accurate when obtained using the first (‘oldest’) observed concentration compared with the most recent observed vancomycin concentration (14). By contrast, in the general patient population, it was reported that taking more TDM data into account did improve the performance of Bayesian forecasting for vancomycin (15).

The median PE values across scenario’s (Figs. 3 and 4) are all negative indicating concentrations are typically underpredicted. We indeed observe a decreasing trend of the random-effects on the CL over time (Fig. 5). Considering that the time-varying covariate creatinine clearance, which is the only potential time-varying covariate identified in most published vancomycin PK models (11,16), was taken into account, a likely explanation is that there could still be other time-varying factors that are difficult to be captured by covariates, e.g. health condition. Patients staying long at the ICU usually require more clinical care indicating a deteriorated health condition and possibly worsening organ function. Such results demonstrated that changes in health condition and related PK in ICU patients may occur and could limit the utility of previously published models for model-based TDM. Our analysis provided a possible solution to best deal with this situation. Clinical practitioners should be well aware of the disease progress of the patient and should primarily take the most recent history into account for Bayesian forecasting. Further improvement of the model is desirable to account time-varying PK, but is not the purpose of this study.

The adaptive MAP method turned out to be most accurate and precise for Bayesian forecasting. A similar approach has been recently proposed for a tacrolimus PK model and it showed that such an approach can be more robust with regard to model misspecification (17). Compared to the standard MAP method, the adaptive MAP method led to a smaller median PE with a smaller accompanying IQR except for including only the preceding one day of historical data. Only one (the first) MAP iteration needs to be executed in such a case, so that the adaptive MAP is identical to the standard MAP (Fig. 3a, b). When including historical data from further past, the adaptive MAP method extracts the information of a segment of historical data in one iteration and carries it forward into the next iteration. Hence, the prior distribution is always updated by the last posterior which is tailored by the historical data from the preceding iteration. After the last iteration, the accumulated information of the historical data was optimally “resembled” through the iterative procedure rather than averaged at once as is done in the standard MAP method. Consequently, the prediction is generated with smarter use of data and thus the accuracy and the precision are improved. Although the most accurate Bayesian forecasting was observed when only including the preceding one day of historical data for the adaptive MAP method, including historical TDM data from the preceding two days is favorable as precision improved drastically with negligible loss in accuracy, which may cause clinically relevant consequences. Provided an actual AUC_0–24_ is 480 mg∙h/L, the IQR of its prediction would be from 382 to 537 mg∙h/L using the preceding one day of TDM data (IQR of PE from −15.2% to 11.9%), while that would be from 443 to 517 mg∙h/L using the preceding two days of TDM data (IQR of PE from −7.7% to 7.7%). As an AUC_0–24_between 400 and 600 mg∙h/L is now recommended the target range for effective and safe vancomycin treatment, the former would unrightfully indicate a dose adjustment while the latter would not (3).

Regarding the weighted MAP method, the underlying hypothesis was that with increasing “age” of the historical data their carried information on the PK of the drug would be of less importance for predicting the data in the future. Mathematically, it equates with weighting the likelihood contribution of each data point to the total likelihood. The time distance between the historical data and the prospective data was chosen to construct the weighting function given our hypothesis. The main reason we additionally added two parameters Δ*T*_*Ref*_ and *α* to the weighting function was to incorporate flexibility in the exploration on how much effect time distance could have on the likelihood as well as on the Bayesian forecasting. Unfortunately, the weighted MAP method showed limited improvement in Bayesian forecasting compared to the standard MAP method. The reason is probably that the weighted MAP method alters the likelihood by relocating the relative importance of data points based on the time distance but does not affect the prior distribution. Given that TDM data are usually sparse, the prior distribution is likely to have a greater impact on the MAP estimation than the data does. Hence, the weighted MAP method is not able to optimize the MAP estimation as much as the adaptive estimation does. We evaluated the values of Δ*T*_*Ref*_ and *α* using only a limited number of integer combinations, but this should not have a harmful impact on the results. For Δ*T*_*Ref*_, there is an obvious interpretability that it refers to the time distance. For *α*, its value is not anticipated to be considerably large or small, which indicates to either diminish the contribution of prior distribution or the likelihood to the MAP estimation. There might be some precision loss not evaluating non-integers, but the result should be covered in the investigated range and the precision may not be much off.

Despite that this study focuses on vancomycin only, the findings are potentially generalizable for other TDM drugs as well. First, the ICU data set used in this study is large. This ensures the ICU patient population is well represented which guarantees the validity of finding. Second, the evidence of the findings in this study is not drug specific. It is ICU patient population rather than vancomycin that results in the observed altering PK properties in the data. It is thus physiologically plausible to assume that similar behaviors may be observed for other drugs in ICU patient. Nonetheless, the applicability of the finding to other TDM drugs in ICU patients needs to be verified and cautions should be taken when making decisions in clinical practice.

Since Bayesian forecasting in routine practice is usually based on the MAP estimation, it inherits the properties as well as the drawbacks of the point estimation. As the PK models used for Bayesian forecasting are usually nonlinear models, the point estimate of MAP does not necessarily relate to the most probable clinical outcome. MAP estimation does not address the uncertainty of the posterior distribution and hence is not able to quantify the potential risk of the relevant clinical outcome. Recently, MAP-based approaches have been questioned in certain circumstances, such as when trough concentration is of interest because even a subtle discrepancy could be damaging for concentrations at a low level (18). Although a full Bayesian approach is superior to MAP-based approaches in handling mentioned issues, the benefit from such an approach could be limited or perhaps clinically irrelevant, due to the relatively large noise in the routine TDM data from ICU patients which influences the results as well. Considering the requirement of extensive domain knowledge in non-clinical fields, a full Bayesian approach is conceivably difficult to be implemented for routine practice and has poor translational ability to communicate to the clinical professionals.

Nonparametric estimation has been proposed and has shown to outperform parametric estimation in the cases where the assumption of normal distribution of the data is violated, while the nonparametric estimation does not make any assumptions as such (19). Indeed, nonparametric approach by theory could be better suited for a particular type of tasks. Yet, it has not been widely adopted in clinical practice and thus the experience is limited in the community. Further investigation and understanding of the differences between parametric and nonparametric approaches and their applicability are certainly required but are out of the scope of this study. Although the parametric MAP-based approaches may have limitations, we believe they are likely to retain the mainstay across the community for a considerable time.

There are limitations worth mentioning regarding this study. First, the creatinine clearance of patients was calculated using the MDRD equation normalized to body surface area. We were not able to calculate the absolute MDRD (which we should ideally have as the original population PK model used measured creatinine clearance) since height (required to estimate body surface area) was not available at the time the data set was obtained. This may affect the descriptive ability of the model for our data set. However, considering that the used model was externally validated using the same data set (11) and MAP estimation can correct individual PK parameters, the consequence of using normalized MDRD should be modest. Second, since vancomycin concentration is usually measured sparsely in routine clinical practice, the impact of the intensity of samples within one dosing interval was not investigated with TDM data in this study. This however may be of interest for other TDM drugs for which samples can be measured multiple times in a dosing interval. Third, we identified that the most recent historical data have best predictive value. Yet, we didn’t investigate what the time distance to the last historical data could be to still have acceptable predictive performance of Bayesian forecasting. This was because the sampling scheme of vancomycin followed the local policy, where trough samples are collected prior to the next dose which are usually scheduled regularly. The time distance to the last historical data is thus usually fixed. Yet, it is our expectation that a greater time distance to the last historical data comes with higher PE, as was also shown by Broeker *et al*. in a mixed patient population receiving vancomycin (14). Last, although the time-varying covariate creatinine clearance was taken into account in the analysis, further inclusion of time-varying characteristics is expected to improve the performance of Bayesian forecasting using the standard MAP method. This can however be challenging due to the need for sufficient data abundancy over the course of therapy. Besides, the inclusion of inter-occasion variability can probably improve the model and also predictive performance of the standard MAP method, but it is often difficult to define occasions in the local data set in the same way as was done for the development of the population PK model.

In conclusion, the adaptive MAP method seems to be promising for model-based TDM of vancomycin in ICU patients with better performance in Bayesian forecasting than the standard MAP method. One should include historical TDM data of the preceding one day when using the standard MAP method or the weighted MAP method, or of the preceding two days when using the adaptive MAP method for the most reliable Bayesian forecasting of vancomycin for ICU patients.

## Electronic supplementary material

ESM 1 (DOCX 216 kb)

## Abbreviations

CL: Clearance
ICU: Intensive care unit
MAP: Maximum a posteriori
NONMEM: Nonlinear mixed-effects modeling
PE: Percentage error
PK: Pharmacokinetic
SD: Standard deviation
TDM: Therapeutic drug monitoring
V: Volume of distribution

## References

[1] Elbers PWG, Girbes A, Malbrain MLNG, Bosman R (2015). Right dose, right now: using big data to optimize antibiotic dosing in the critically ill. Anaesthesiology intensive therapy.

[2] Monteiro JF, Hahn SR, Gonçalves J, Fresco P. Vancomycin therapeutic drug monitoring and population pharmacokinetic models in special patient subpopulations. Pharmacol Res Perspect. 2018 Aug 28;6(4):e00420. 10.1002/prp2.420 PMC6113434 30156005

[3] Rybak MJ, Le J, Lodise TP, Levine DP, Bradley JS, Liu C, *et al*. Therapeutic monitoring of vancomycin for serious methicillin-resistant *Staphylococcus aureus* infections: A revised consensus guideline and review by the American Society of Health-System Pharmacists, the Infectious Diseases Society of America, the Pediatric Infectious Diseases Society, and the Society of Infectious Diseases Pharmacists. Am J Health Syst Pharm. 2020 May 19;77(11):835-864. 10.1093/ajhp/zxaa036 32191793

[4] Liu C, Bayer A, Cosgrove SE, Daum RS, Fridkin SK, Gorwitz RJ, *et al.* Clinical practice guidelines by the Infectious Diseases Society of America for the treatment of methicillin-resistant Staphylococcus aureus infections in adults and children. Clin Infect Dis. 2011 Feb 1;52(3):e18–55. 10.1093/cid/ciq146 21208910

[5] Ye Z-K, Chen Y-L, Chen K, Zhang X-L, Du G-H, He B, *et al.* Therapeutic drug monitoring of vancomycin: a guideline of the division of therapeutic drug monitoring, Chinese Pharmacological Society. J Antimicrob Chemother. 2016 Nov 1;71(11):3020–5. 10.1093/jac/dkw254 27494905

[6] Matsumoto K, Takesue Y, Ohmagari N, Mochizuki T, Mikamo H, Seki M, *et al.* Practice guidelines for therapeutic drug monitoring of vancomycin: a consensus review of the Japanese Society of Chemotherapy and the Japanese Society of Therapeutic Drug Monitoring. J Infect Chemother. 2013 Jun;19(3):365–80. 10.1007/s10156-013-0599-4 23673472

[7] Roggeveen LF, Guo T, Driessen RH, Fleuren LM, Thoral P, van der Voort PHJ, *et al*. Right Dose, right now: Development of autokinetics for real time model informed precision antibiotic dosing decision support at the bedside of critically Ill patients. Front Pharmacol. 2020 May 15;11:646. 10.3389/fphar.2020.00646 PMC7243359 32499697

[8] Levey AS, Bosch JP, Lewis JB, Greene T, Rogers N, Roth D (1999). A more accurate method to estimate glomerular filtration rate from serum Creatinine: a new prediction equation. Ann Intern Med.

[9] Bragadottir G, Redfors B, Ricksten S-E (2013). Assessing glomerular filtration rate (GFR) in critically ill patients with acute kidney injury--true GFR versus urinary creatinine clearance and estimating equations. Crit Care.

[10] Roberts JA, Taccone FS, Udy AA, Vincent JL, Jacobs F, Lipman J (2011). Vancomycin dosing in critically ill patients: robust methods for improved continuous-infusion regimens. Antimicrob Agents Chemother.

[11] Guo T, van Hest RM, Roggeveen LF, Fleuren LM, Thoral PJ, Bosman RJ, *et al.* External evaluation of population pharmacokinetic models of Vancomycin in large cohorts of intensive care unit patients. Antimicrob Agents Chemother. 2019 May 1;63(5):e02543–18. 10.1128/AAC.02543-18 PMC6496102 30833424

[12] Sheiner LB, Beal SL (1982). Bayesian individualization of pharmacokinetics: simple implementation and comparison with non-Bayesian methods. J Pharm Sci.

[13] Wang L. Model predictive control system design and implementation using MATLAB®. London: Springer-Verlag; 2009. (Advances in Industrial Control).

[14] Broeker A, Nardecchia M, Klinker KP, Derendorf H, Day RO, Marriott DJ, *et al*. Towards precision dosing of vancomycin: a systematic evaluation of pharmacometric models for Bayesian forecasting. Clin Microbiol Infect. 2019 Oct;25(10):1286.e1–1286.e7. 10.1016/j.cmi.2019.02.029 30872102

[15] Deng C, Liu T, Wu K, Wang S, Li L, Lu H, *et al.* Predictive performance of reported population pharmacokinetic models of vancomycin in Chinese adult patients. J Clin Pharm Ther. 2013;38(6):480–9. 10.1111/jcpt.12092 24033587

[16] Cunio CB, Uster DW, Carland JE, Buscher H, Liu Z, Brett J, *et al*. Towards precision dosing of vancomycin in critically ill patients: an evaluation of the predictive performance of pharmacometric models in ICU patients. Clinical Microbiology and Infection. 2020 Jul 13;S1198-743X(20)30388-8. 10.1016/j.cmi.2020.07.005 32673799

[17] Ruben Faelens, Nicolas Luyckx, Quentin Leirens, Dirk Kuypers, Thomas Bouillon. Model predictive control with Bayesian updates (MPC) is more robust to model misspecification, compared to standard Bayesian control (sEBE) for Therapeutic Drug Management (TDM). Investigation in a cohort of 315 patients receiving tacrolimus during the first 14d after renal transplantation. In PAGE 28 (2019); 2019. Available from: https://www.page-meeting.org/default.asp?abstract=9076.

[18] Maier C, Hartung N, de Wiljes J, Kloft C, Huisinga W (2020). Bayesian data assimilation to support informed decision making in individualized chemotherapy. CPT Pharmacometrics Syst Pharmacol.

[19] Tatarinova T, Neely M, Bartroff J, van Guilder M, Yamada W, Bayard D, *et al.* Two general methods for population pharmacokinetic modeling: non-parametric adaptive grid and non-parametric Bayesian. J Pharmacokinet Pharmacodyn. 2013 Apr;40(2):189–99. 10.1007/s10928-013-9302-8 PMC3630269 23404393

