# Correction to: Optimizing Predictive Performance of Bayesian Forecasting for Vancomycin Concentration in Intensive Care Patients

**DOI:** 10.1007/s11095-020-02934-5

**Published:** 2020-10-19

**Authors:** Tingjie Guo, Reinier M. van Hest, Laura B. Zwep, Luca F. Roggeveen, Lucas M. Fleuren, Rob J. Bosman, Peter H. J. van der Voort, Armand R. J. Girbes, Ron A. A. Mathot, Paul W. G. Elbers, Johan G. C. van Hasselt

**Affiliations:** 1 Department of Intensive Care Medicine | Research VUmc Intensive Care (REVIVE) | Amsterdam Cardiovascular Sciences (ACS) | Amsterdam Medical Data Science (AMDS), Amsterdam UMC, Vrije Universiteit Amsterdam, Amsterdam, The Netherlands; 2 Department of Pharmacy, Amsterdam UMC, University of Amsterdam, Amsterdam, The Netherlands; 3 Division of Systems Biomedicine and Pharmacology, Leiden Academic Centre for Drug Research (LACDR), Leiden University, Leiden, The Netherlands; 4 Mathematical Institute, Leiden University, Leiden, The Netherlands; 5 Intensive Care Unit, OLVG Oost, Amsterdam, The Netherlands

**Pharm Res (2020) 37: 171**

10.1007/s11095-020-02908-7

This article was updated to correct Figs. 1 and 4 as author corrections were overlooked during the production process.

**Fig. 1:**
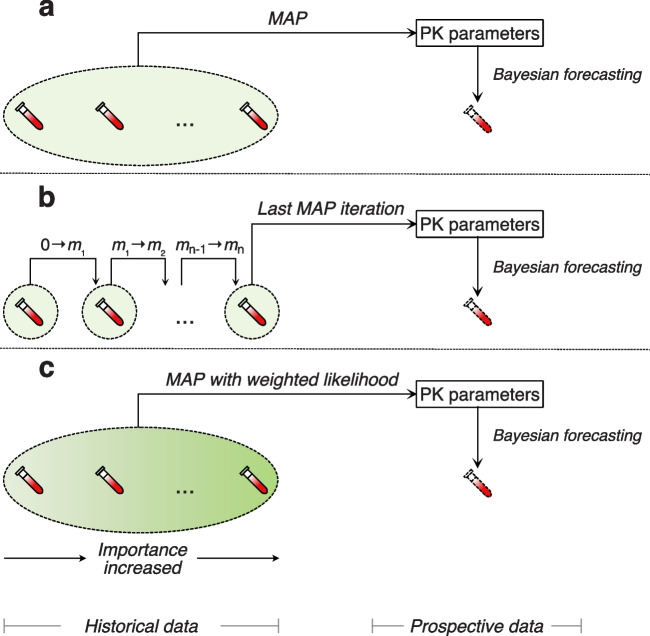
Schematic diagram of methods of MAP estimation used in the study. Standard MAP executes estimation once using all historical TDM data (**a**); Adaptive MAP executes estimation iteratively using each segment of historical TDM data with updated prior mean by posterior mode from its preceding iteration and repeats until the last iteration, i.e. 0 to m1, …, mn-1 to mn (**b**). Weighted MAP executes estimation once using all historical TDM data with weighted importance (likelihood) of each segment of data during the estimation (**c**).

**Fig. 4:**
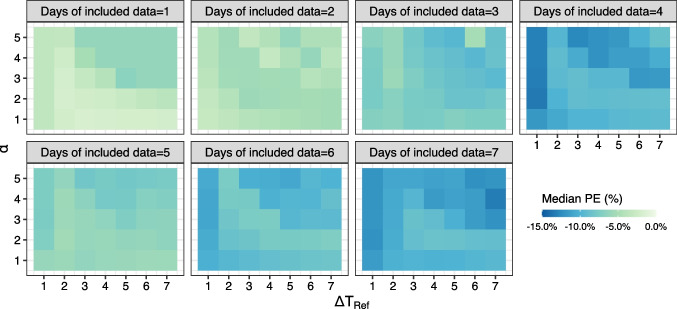
The percentage error of Bayesian forecasting using the weighted MAP method for all combinations of weighting factors ΔTRef and α. ΔTRef and α are both weighting factors. ΔTRef is the reference day, defined as the cutoff value of the time distance where w(Ys) is 1, i.e.where ΔTRef equals ΔT Ys . α is the unitless effect size of the weighting function w(Ys) on the likelihood f(η| Y).

