# Supplementary material for: Optimizing Predictive Performance of Bayesian Forecasting for Vancomycin Concentration in Intensive Care Patients
Source: Pharm Res. 2020 Aug 23;37(9):171. doi: 10.1007/s11095-020-02908-7 (PMC7443423; doi:10.1007/s11095-020-02908-7)
Supplement: Supplementary file 1 — (DOCX 216 kb) [file 11095_2020_2908_MOESM1_ESM.docx]

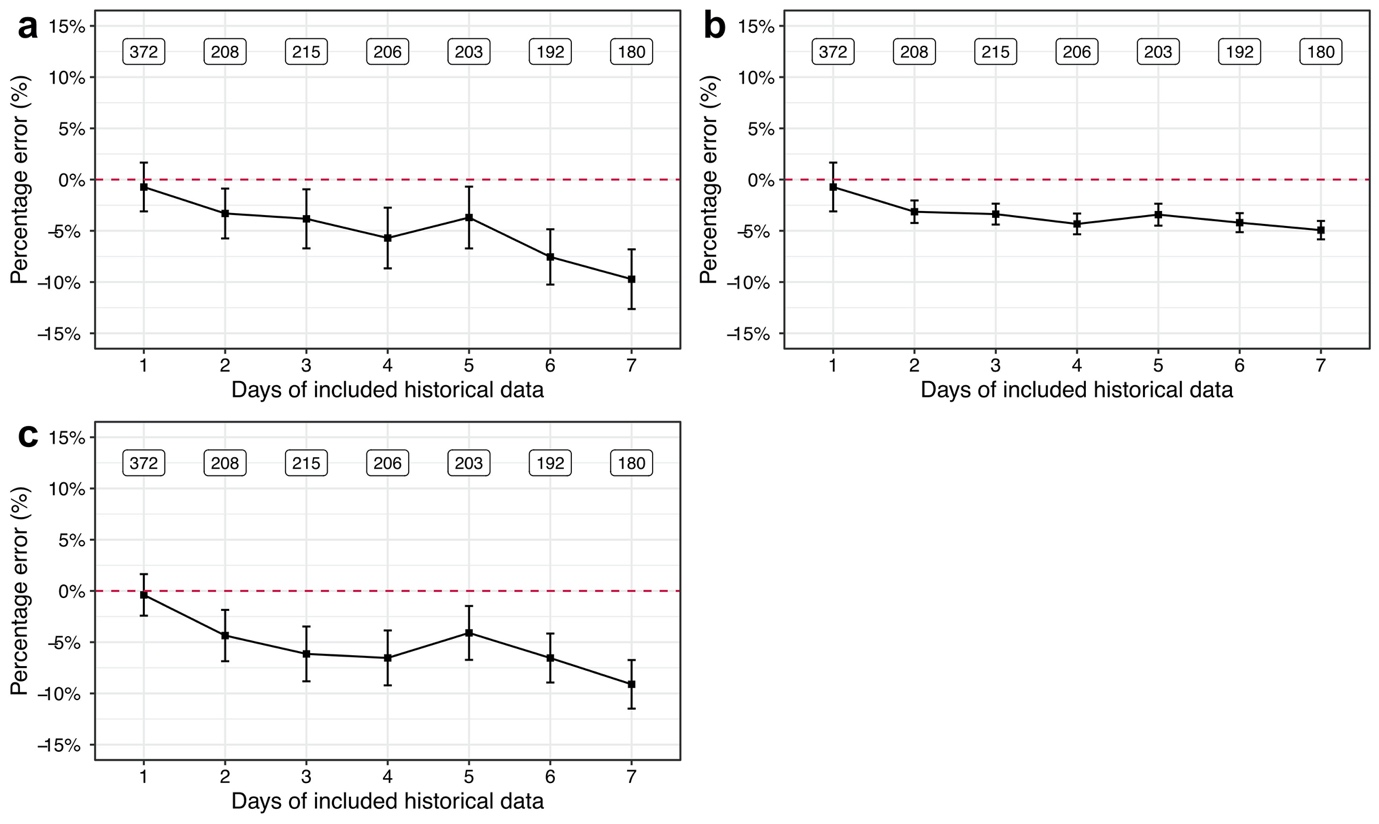


**Figure S1**. The percentage error of Bayesian forecasting using the standard MAP method (a), the adaptative MAP method (b), and the weighted MAP method using optimal weighting factors $\Delta T_{Ref}$=4 and $\alpha$=2 (c). The squares are the mean values, and the bars represent lower and upper boundaries of 95% confidence interval. The labeled text is the number of patients that were included for the calculation.

# NONMEM control stream

## *Adaptive MAP estimation (First iteration)*

$PROBLEM PK

$INPUT C DROP ID TIME DROP AMT RATE ADDL II CMT DV EVID AGE SEX WGT MDRD DROP OCCDAYSN DROP SCE SINGLE ETACL ETAV

$DATA ./LN_prior_1.csv IGNORE=C IGNORE=(OCCDAYSN.GT.1)

$SUBROUTINES ADVAN1 TRANS2

$PK

TVCL = THETA(1) * MDRD/100

TVV = THETA(2) * WGT

IF(NEWIND.NE.2) THEN

OETACL = ETACL

OETAV = ETAV

ENDIF

CL = TVCL * EXP(OETACL + ETA(1))

V = TVV * EXP(OETAV + ETA(2))

OETACL = ETACL

OETAV = ETAV

S1 = V

$ERROR

IPRED = F

W = 1

IRES = DV - IPRED

IWRES = IRES/W

Y = IPRED + IPRED*EPS(1) + EPS(2)

$THETA

4.58 ; TVVL (L/h)

1.53 ; TVV (L/kg)

$OMEGA

0.151321 ; omega^2 CL

0.139876 ; omega^2 V

$SIGMA

0.039601 ; Prop

5.76 ; Add

$EST METHOD=1 INTER MAXEVAL=0 NOABORT SIG=3 PRINT=1

$TABLE ID EVID ETA(1) ETA(2) ONEHEADER NOPRINT FILE=eta_LN_prior_1.tab

## *Weighted MAP estimation*

$PROBLEM PK

$INPUT C OID ID TIME DROP AMT RATE ADDL II CMT DV EVID AGE SEX WGT MDRD OCCDAY DROP DROP SCE SINGLE MAXOCCDAY

$DATA ./LN_weight.csv IGNORE=C IGNORE=(C==2) IGNORE=(SINGLE==1)

$SUBROUTINES ADVAN1 TRANS2

$PK

TVCL = THETA(1) * MDRD/100

TVV = THETA(2) * WGT

CL = TVCL * EXP(ETA(1))

V = TVV * EXP(ETA(2))

S1 = V

$ERROR

YHAT = F

VARA = 5.76

VARP = 0.039601

VAR = VARA + VARP*YHAT**2

REF = 1

A = 1

W = REF/(MAXOCCDAY-OCCDAY)

Y = (1/SQRT(2*3.1415926*VAR)*EXP(-(DV-YHAT)**2/(2*VAR)))**(W**A)

$THETA

4.58 FIX ; TVVL (L/h)

1.53 FIX ; TVV (L/kg)

$OMEGA

0.151321 FIX ; omega^2 CL

0.139876 FIX ; omega^2 V

$EST METHOD=COND LAPLACE LIKE MAXEVAL=0 NOABORT PRINT=1

$TABLE ID EVID SINGLE SCE W ETA(1) ETA(2) ONEHEADER NOPRINT FILE=eta_r1a1.tab
